# Rabies epidemiology, prevention and control in Nigeria: Scoping progress towards elimination

**DOI:** 10.1371/journal.pntd.0009617

**Published:** 2021-08-16

**Authors:** Philip P. Mshelbwala, J. Scott Weese, Olufunmilayo A. Sanni-Adeniyi, Shovon Chakma, Stephen S. Okeme, Abdullah A. Mamun, Charles E. Rupprecht, R. J. Soares Magalhaes

**Affiliations:** 1 UQ Spatial Epidemiology Laboratory, School of Veterinary Science, University of Queensland, Gatton, Australia; 2 Department of Veterinary Medicine, University of Abuja, Abuja, Nigeria; 3 Department of Pathobiology, Ontario Veterinary College, Guelph, Canada; 4 Federal Ministry of Health, Abuja, Nigeria; 5 Agriculture & Rural Development Secretariat, Federal Capital Territory Administration Abuja Nigeria; 6 Institute of Social Science Research, the University of Queensland, Long Pocket, Australia; 7 Rabies Section, LYSSA LLC, Cumming, Georgia, United States of America; 8 Children’s Health and Research Centre, Children’s Health and Environment Program, the University of Queensland, South Brisbane, Australia; Saudi Ministry of Health, SAUDI ARABIA

## Abstract

**Background:**

Human rabies remains a significant public health problem in Africa with outbreaks reported in most countries. In Nigeria–the most populous country in Africa–rabies causes a significant public health burden partly due to perennial obstacles to implementing a national prevention and control program.

**Methods:**

We conducted a scoping review using standard Preferred Reporting Items for Systematic Reviews and Meta-Analyses (PRISMA) guidelines to identify and select published articles from Nigeria during 1978–2020 reporting on rabies virus infections (human, canine, livestock, and wildlife), canine bites, knowledge, attitudes and practices (KAP) surveys on rabies and canine ecology studies. We extracted information on study location, year and additional details of each study such as rabies prevalence, general characteristics of offending dogs, dog vaccination status and health-seeking behaviours.

**Findings:**

Between 1978 and 2020, 90 published articles met our inclusion criteria. The prevalence of rabies virus antigen detection varied between 3% and 28%, with more studies in the north. Most bites were unprovoked from dog bite studies (36.4%-97%), by dogs with low vaccination rates (12–38%). A more significant proportion of biting dogs were owned (31–90%). Laboratory confirmation for biting was available for only a small proportion of studies (6%; n = 2/32). Of the dogs surveyed during ecology studies, indigenous dogs accounted for the majority (62–98%), used mostly for security purposes (52–98%), with the vaccination rate between 15% and 38% in most states. Studies conducted in areas distant from rabies diagnostic facilities accounted for more human rabies cases and fewer dog rabies cases.

**Conclusion:**

Significant improvements are necessary to achieve the elimination of human rabies mediated via dogs by 2030.

## Author summary

Rabies remains one of the deadliest zoonoses known to humanity since antiquity, resulting in tens of thousands of human deaths each year, mostly in African and Asian countries. Mass dog vaccination, enhanced surveillance, improved access to Pre-exposure prophylaxis(PrE) and (Post-exposure prophylaxis(PEP) to at-risk groups, enforcement of responsible dog ownership and public education are keystones to rabies prevention and control. Since the first reported case in Nigeria during 1912, rabies has continued to take its toll on human and animal lives. However, gross underreporting due to a frail health care infrastructure, deficient reporting systems, and misdiagnosis with neurological diseases further complicated by socio-cultural practices have resulted in a lack of empirical data to support rabies prioritization, per the global target of 2030. We used the standard PRISMA guidelines to select Nigerian rabies studies between 1978 and 2020. Rabies risk, coupled with welfare concerns identified in this study, demonstrates the need for continued advocacy towards legislation prohibiting the dog trade in Nigeria and elsewhere. The high-risk community groups identified in this review (i.e., children, butchers, and adult males) need to be educated on response to dog bite exposure to reduce the impact of this invariable fatal but preventable disease. Moreover, there is a need for mandatory dog vaccination and implementation of a national rabies program to attain the World Health Organization (WHO) recommended vaccination coverage of at least 70%. We unravel the need to establish rabies diagnostic centres in the country’s six geopolitical zones. Local inclusion of dog bite occurrence by Disease Surveillance and Notification Officers (DSNOs), with related community surveillance tools, can help in rabies/dog surveillance. In addition, there is a need for investment into pathogen discovery by enhancing laboratory-based surveillance for wildlife rabies, understanding its potential role in Nigeria and the need for quantitative research to understand the various risk factors for disease perpetuation.

## Introduction

Rabies is a fatal and progressive zoonotic neurological disease caused primarily by rabies virus RV, a member of the Lyssavirus genus (in the family Rhadoviridae of the order Mononegavirales) affecting all warm-blooded animals, mainly transmitted through bites from rabid animals [1]. In developing countries, 99% of human rabies deaths are due to bites from rabid dogs [2,3]. Unfortunately, data on dog bite injuries and related mortality are fragmented in most developing countries [4]. However, in regions that have successfully eliminated dog rabies, wildlife such as bats, ferret badgers, foxes, mongoose, raccoons, raccoon dogs, and skunks are significant RV reservoirs [5]. Rabies has the highest case fatality of any infectious disease [6]. Prompt and proper human PEP is highly effective if administered before the illness. Globally, tens of thousands of people are estimated to die from rabies each year (95% Cl: 25,000–159,000) [3,7]. Although effective vaccines are available for humans and animals, rabies is still estimated to result in about 3.7 million years of life lost and $8.6 billion economic losses per year [3].

During 2015, the World Health Organization (WHO), the Food and Agriculture Organization (FAO), the World Organization Animal Health (OIE) and the Global Alliance for Rabies Control (GARC) set a global call for action against rabies. The goal was to target zero human deaths from dog-mediated rabies by 2030 [8]. Dog vaccination, provision of PEP to exposed persons, enhanced laboratory-based surveillance, PrEP vaccination of at-risk groups, coupled with educational outreach to improve community knowledge, are the cornerstones to rabies prevention and control [9]. During 2014, GARC started the Pan-African Rabies Control Network (PARACON), with an inaugural meeting on 9–11 June 2015 in South Africa [9]. The aim was to create a consolidated One Health network for rabies management throughout the continent, using available evidence to plan and support rabies prevention, control and eventual canine rabies elimination in sub-Saharan Africa [10]. Individual countries were encouraged to develop national rabies elimination strategies using the stepwise approach[10]. The collection of epidemiological information to estimate the burden of rabies is a key in the stepwise method. Kenya and Namibia have created and begun implementing a plan through large-scale dog rabies vaccination, resulting in a decline in human deaths[11,12]. Before implementation, evidence of the burden of rabies obtained countrywide helped in the prioritisation of interventions.

Nigeria comprises 36 states and the Federal Capital Territory, Abuja within West Africa and borders the Republic of the Niger in the north, Chad in the north-west, Cameroon in the east and Benin in the west. Nigeria is the most populous country in Africa and the seventh most populous country globally, with an estimated population of 208,580,545 as of December 2020 (based on United Nations data) [13]. A total of 9,234 veterinarians were registered with the Veterinary Council of Nigeria as of 2019 [14]. Of these, only 11% are public veterinarians (i.e., employed by the government), with Yobe State in the northeast having the largest number (i.e., 126/1088) of public veterinarians, while Rivers State (south-south) has only one veterinarian [14]. Since the first reported human case during 1912, rabies has remained a significant public health problem with numerous reports in humans and animals [15,16]. Thousands of people are estimated to die from rabies each year in Nigeria [17]. Nigeria carried out the last mass dog vaccination campaign during 1982[15]. During a recent global virtual event (United Against Rabies Forum, 22–23 September 2020), the Nigerian representative highlighted challenges concerning data gaps and inadequate funding as some of the obstacles to implementing a national control program (https://uarforum.org/). The lack of reliable and sustained information is a considerable challenge for future rabies prevention, control and elimination [18].

In Nigeria, anecdotal reports on dog bites abound in human clinics and veterinary hospitals, but only a minority are documented [19,20]. Moreover, routine RV diagnosis is only available at the National Veterinary Research Institute (NVRI), Vom, Nigeria [21]. Other institutions carry out testing for research studies. Concerning prophylaxis, one recent study found that PEP is not readily available in hospitals across the country [22]. In contrast, reports on dog slaughter for consumption abound, with potential for RV infection, especially among individuals involved in the chain of capture, breeding, purchase, transportation, slaughtering, processing and handling [23,24]. Despite these reports of continuing RV transmission and deaths, a rabies elimination strategy has not been implemented to date [25]. In the light of the existing sociodemographic and health service challenges, there is no consolidated information about the rabies incidence or understanding the key factors driving RV transmission in the country, prompting challenges in current efforts towards a national control strategy. A thorough understanding of rabies epidemiology is necessary for effective planning and implementation of prevention and control. Effective planning of rabies vaccination programs using baseline epidemiological data is crucial to attaining the 2030 goal of zero dog-mediated human rabies deaths in Nigeria.

In this scoping review, we retrieved published literature on rabies from Nigeria to establish an evidence-base with regards to currently incomplete knowledge on the prevalence of dog, livestock and wildlife rabies; the frequency of dog bites events and their relationships with the occurrence of human rabies; the extent of community knowledge, attitude and practices (KAP) surveys and relevant dog applied ecology and demographic patterns. Our primary objective was to summarise existing data to help support the design of a national rabies action plan towards attaining the target of zero human deaths from dog rabies by 2030.

## Materials and methods

### Literature search strategy

As a precursor to more in-depth systematic analyses, scoping reviews are useful to provide a preliminary overview of a problem regarding key concepts, available studies, and associated research gaps [26]. We used the PRISMA guidelines to search and identify relevant papers[27]. Our search priority was to identify published articles on rabies in animals (i.e. dogs, livestock, wildlife, and other animals) and humans in Nigeria. Keywords were ’rabies’ OR "dog bite" AND ’dog ecology’ AND ’dog demography’ AND ’Nigeria’. We applied the search strategy to the following scientific literature databases between June and August 2020: PubMed, Web of Science, Scopus, Google scholar, and complemented by emails to researchers. We stored all articles using the reference manager EndNote (Thomson Reuters, Philadelphia, PA, USA) and removed duplicates. Additional relevant studies were searched manually from the reference list of included articles.

### Study selection

**Fig 1:**
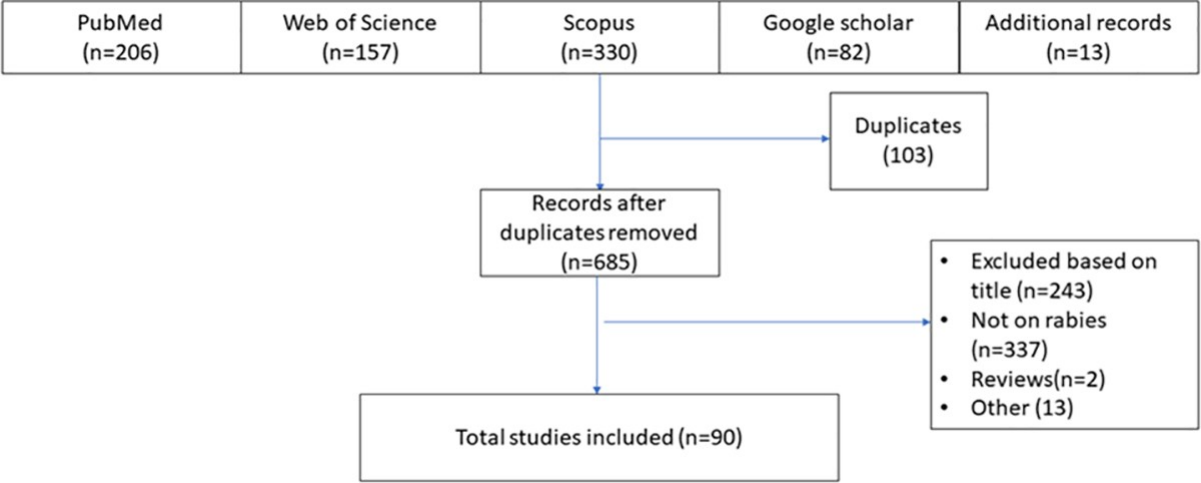
Search and selection strategies based on PRISMA guidelines.

The corresponding author (Philip P. Mshelbwala) independently selected the studies. First, titles and abstracts were reviewed initially (Fig 1). If a decision on the inclusion/exclusion based on the title and abstract was insufficient, the full article was reviewed. Original full articles and case reports were eligible for review if they met the following criteria: Firstly, rabies studies (canine, livestock and wildlife) including case reports and conference proceedings; secondly dog ecology and demographic studies; thirdly dog bite studies in human and veterinary health care facilities and the relationship with human rabies occurrence; and KAP studies on rabies between 1978 and 2020. If data were unclear or incomplete, we contacted the authors of original publications for clarification. Of the thirteen authors contacted, eight responded.

### Data extraction

We developed a standard extraction form to capture relevant information for the critical appraisal of each study. For all studies, we extracted the year of the study and study location. For studies on dog rabies, we retrieved the following information: the source of the sample, type of test conducted, the outcome of the laboratory test, information on the market chain of dogs for human consumption (e.g., mode of transportation), the practice of the dog butcher, and serological results of butchers, consumers and veterinarians (when available). For studies that reported dog bite occurrence, we extracted the following: the gender of the victim, site of the bite, whether the victim sought PEP, vaccination status of the offending dog, season and the reason for the dog bite report (e.g., provoked vs unprovoked). For dog ecology studies, we extracted the following information: study setting, dog vaccination status, the purpose of keeping dogs, dog breed and dog-human ratio. For KAP studies, we looked at the target population, study location, and KAP scores related to rabies. Finally, for livestock and wildlife rabies studies, we extracted information on the species affected, the lyssavirus species or RV variant in circulation, and the study’s location. Finally, we searched for information on current prevention and control efforts from experts in the field and information online.

## Results

### Characteristics of studies included in the review

**Fig 2:**
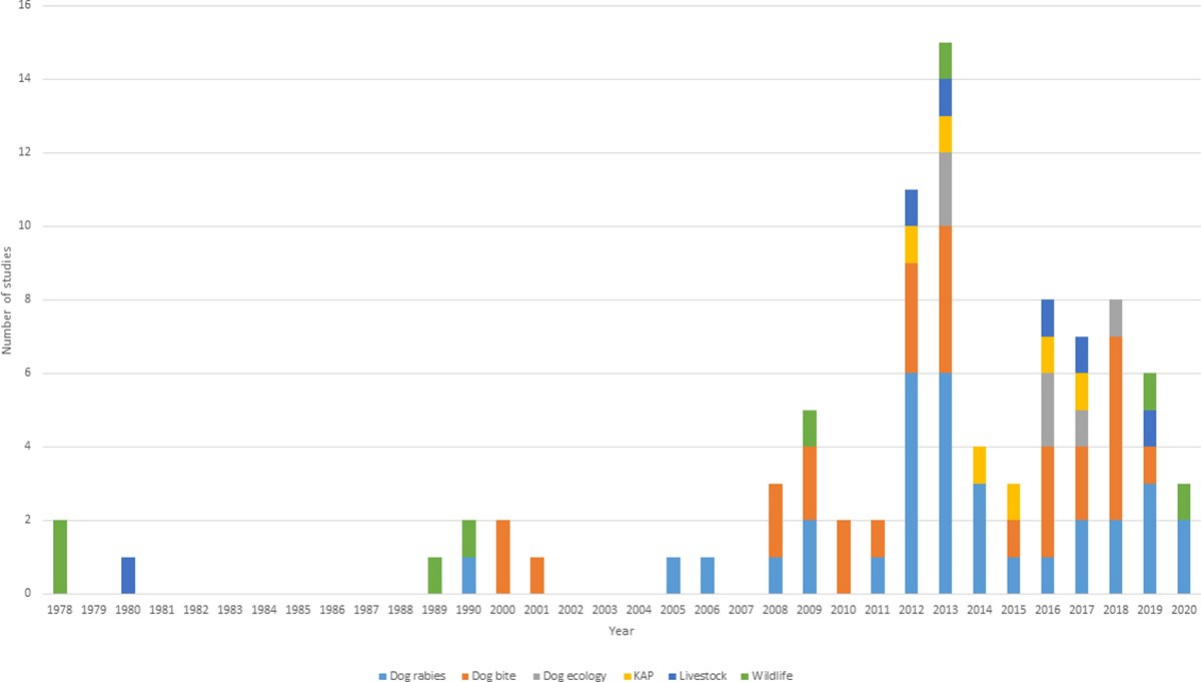
Number of selected studies by year and type between 1978 and 2020.

Between 1978 and 2020, 90 studies met our inclusion criteria (Fig 1). A total of 33% (n = 30/90) of studies reported dog bite incidence, 36% (n = 32/90) reported prevalence of RV diagnostic detection among dogs slaughtered for human consumption, 9% (n = 8/90) were dog ecology studies, 7% (n = 6/90) reported community KAP surveys, 8% (n = 7/90) were studies on rabies in livestock and 8% (n = 7/90) were studies on wildlife rabies. Between 1978 and 1989, there were more studies on livestock and wildlife rabies, and after 1990 there was an increase in the number of studies reporting dog rabies and dog bite studies. Starting from 2012 until 2018, an increase occurred in studies into rabies KAP and dog ecology. Between 1999 and 2019, there was a steady increase in the number of studies on dog rabies, with a peak during 2013 (Fig 2).

### Dog rabies studies

**Fig 3:**
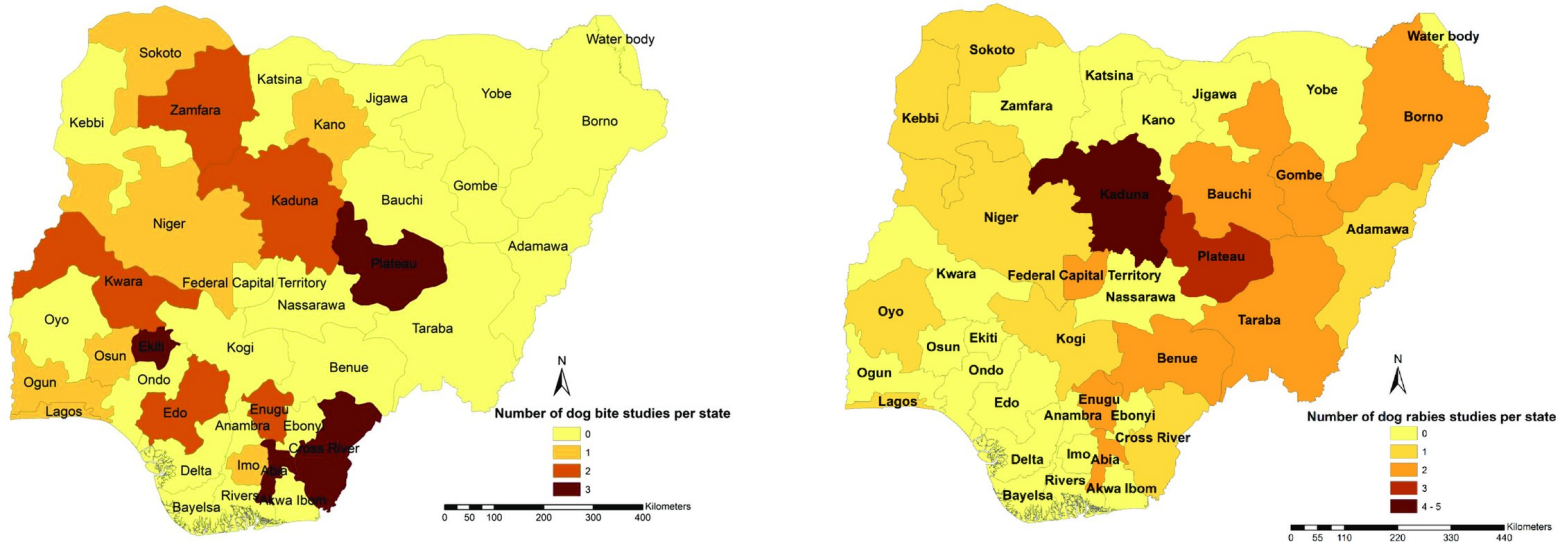
Spatial distribution selected dog bite and dog rabies studies across Nigerian states. The map was created using ArcMap software (ESRI Inc., Redlands, CA, U.S.A.). The shapefile was retrieved from DIVA- GIS (https://www.diva-gis.org/).

A total of 32 studies published between 1999 and 2019 evaluated the prevalence of RV antigen detection among dogs. The majority of these studies, were conducted in the northern region, with 28% (9/32) in the north-central part, 28% (9/32) in the northeast region, and 22% (7/32) in the north-west region, 13% (4/32) in the south-east, 6% (2/32) in the south-west and 3%(1/32) in the south-south (Fig 3).

#### Target population and samples

The majority of brain samples processed in dog rabies studies were collected from dogs meant for slaughter, except for four studies that sampled a combination of dogs intended for meat and dogs with clinical signs indicative of rabies presented to veterinary hospitals[28–31].

#### Laboratory case-definition protocols

Most samples were subjected to the fluorescent antibody test (FAT) (29/32 of studies), and a combination of the mouse inoculation test (MIT), the direct rapid immunohistochemistry test (dRIT), the rapid immunochromatography test (RICT) and the Reverse Transcriptase Polymerase Chain Reaction (RT-PCR) Table 1. A minority of studies reported in-situ hybridisation, immunohistochemistry, histology (Seller’s stain), and the dRIT only[28,31,32].

#### Prevalence and molecular epidemiology of dog rabies

The prevalence of rabies was 6% in the south-south region, between 5% (5/100) and 9% (23/252) in the south-east, 2% (7/444) and 11% (5/47) in the south-west, 4% (6/154) and 50% % (15/30) in the north-west, 5% (10/203) and 17% (92/532) in the north-central and 2% (3/150) and 44% (22/50) in the north-east Table 1. There was an increase in the number of studies on dog rabies and samples processed across the study years, with a peak during 2013. Phylogenetic analysis of RV sequences demonstrated that isolates belong to the African 2 clade [33]. Transboundary transmission of RV was evident, with studies reporting related viral variants to neighbouring countries (e.g., Chad and the Republic of the Niger) [33,34].

### Studies reporting dog bite cases

Between 2000 and 2019, 30 studies reported dog bite occurrence in either veterinary clinics/hospitals, 27% (8/30) and human hospitals/ health care centres 73% (22/30). Of these, 80% (24/30) recorded mortality in humans with a presumptive or confirmed diagnosis of rabies (S1 Table). Cases of human deaths due to rabies were more concentrated in the south-west and south-east regions, especially in Ekiti 7.1% (6/84) - 27.3% (12/44) and Imo 4% (17/436–6.4% (7/110), while studies in other locations reported deaths between 0.5% (1/196) - 11% (5/44) (S1 Table). Human hospital studies generally reported more bites among children (12% (17/143) - 100% (174/174) compared to veterinary hospital studies, where only a minority of 12% (25/215) - 35% (69/196) were affected, except one study that reported 57% (104/183) of bites in children [35,36]. One study reported use of water and soap as first aid by victims of dog bite [37], while 27% (8/30) of studies reported the administration of traditional treatments [16,21,38–43], such as consumption of the abdomen of the biting dog in Cross River [43], use of herbs and concoctions in Abia State [23], and consumption of the cooked liver of stray dogs and placing pulled hair from the dog’s neck on the bite wound in Kaduna [16]. Males reported more bites 47% (92/196) - 95% (36/38) compared to females 19% (15/81) - 44% (66/149). Most victims in Ekiti State 82% (69/84) reported to the hospital within 24 hours of attack [42], similar to reports from Benin 53% (76/143) and Ibadan 87 (869/1000) [40,44]. One study reported delays between 30 and 60 days [40]. Most victims in dog bite studies received PEP (Range: 86% (64/74)– 96% (45/47)). Three studies reported incomplete PEP courses in 4% (6/143) of individuals in Benin, 36% (30/84) in Ekiti, and 50% (499/1000) in Ibadan [40,44,45]. Owned dogs formed a more significant proportion of the biting dog population 44% (63/143) - 90% (223/247) than free-ranging animals 10% (24/223) - 64% (28/44). Most bites were unprovoked 36% (4/11) - 97% (168/174) with low vaccination rates 1% (1/84) - 38% (23/74) of biting animals. Three studies reported bites during the dry season (October–March) [44,46,47] and during the rainy season, between January and April in Niger and November [48] and December in Abia [49]. Laboratory confirmation information for offending dogs was available for only two veterinary hospital studies [36,38].

**Table 1:** Summary of studies reporting the prevalence of rabies virus detection among Nigerian dogs.

SN	State	Year	Type of test	Total sampled	# positive	Prevalence	Ref
1	Abia	2013	FAT, dRIT and RICT (NVRI)	100	5	5%	[23]
2	Abia	2014	FAT (ABU)	185	13	7%	[49]
3	Enugu	2015	FAT (NVRI)	152	6	4%	[103]
4	Enugu	2018	FAT, RT-PCR, (NVRI & Abroad)	252	23	9%	[104]
5	Kogi	2020	FAT (ABU)	208	11	5%	[19]
6	Plateau	2018	FAT & RT-PCR (NVRI & Abroad)	532	92	17%	[33]
7	Lagos	2013	FAT (ABU)	444	7	2%	[30]
8	Adamawa	2010	FAT and Microscopy (NVRI)	50	22	44%	[105]
9	Bauchi	2014	FAT (NVRI)	202	44	22%	[95]
10	Sokoto	2010	FAT and MIT(NVRI)	50	13	26%	[106]
11	Borno	2006	Microscopy and Immunohistochemistry	52	16	31%	[32]
12	Kaduna	2017	FAT & RICT (NVRI)	50	8	16%	[29]
13	Kaduna	2014	FAT, dRIT, RT-PCR and MIT (NVRI and abroad)	30	15	50%	[107]
14	Kaduna	2012	FAT (ABU)	200	14	7%	[108]
15	Kaduna	2011	FAT (ABU)	100	6	6%	[98]
16	Kaduna	2019	FAT (ABU)	154	6	4%	[59]
17	Cross River	2013	FAT (ABU)	177	6	3%	[80]
18	Taraba	2014	FAT (ABU)	188	15	8%	[57]
19	Taraba	2019	FAT (NVRI)	150	3	2%	[109]
20	Abuja	2012	FAT (NVRI)	50	50	100%?	[110]
21	Abuja	2014	FAT (ABU)	150	18	12%	[111]
22	Gombe	2020	FAT and RT-PCR (ABU)	50	3	6%	[81]
23	Oyo	2016	dRIT (UI)	47	5	11%	[28]
24	Gombe	2019	FAT (NVRI)	118	9	8%	[112]
25	Borno	1999	In situ hybridisation (Abroad)	25	11	44%	[31]
26	Benue	2009	FAT (NVRI)	76	12	16%	[113]
27	Plateau	2015	FAT (NVRI)	203	10	5%	[24]
28	Benue	2020	FAT(ABU)	464	52	11%	[114]
29	Kebbi	2019	FAT and RT-PCR (ABU)	49	6	12%	[115]
30	Plateau	2013	FAT (NVRI)	321	150	47%	[116]
31	Niger	2015	FAT (NVRI)	471	4	1%	[117]
32	Bauchi	2007	FAT (NVRI)	44	10	22%	[118]

ABU- Ahmadu Bello University, dRIT- Direct Rapid Immunohistochemistry Test, FAT- Fluorescent Antibody Test, MIT–Mouse Inoculation Test, NVRI- National Veterinary, RT-PCR- Reverse Transcription Polymerase Chain Reaction

### Studies on dog vaccination coverage and population structure

Eight studies reviewed reported estimates of dog vaccination coverage and population structure (Table 2). The majority (99%) of studies reported vaccination rates of 15–66%, well below the WHO recommendation of 70%, except a survey from the Niger State (70%) [50]. One study did not report vaccination rates [51]. In a study of 150 healthy dogs in Nigeria, no dog had an adequate serological titre (e.g., at or above 0.5 IU/ml) [52]. Another study that looked at the titre level in free-roaming (but owned) and stray (dogs without owners) dogs found only 38% and 7% had titres >0.5 IU/ml, respectively [53] using the indirect ELISA. Two studies in Lagos and Abuja found that veterinarians administered 91% and 81% [54,55] of vaccines to dogs, with vaccines also administered by dog owners and veterinary technologists. Studies that attempted to understand vaccination rates among urban/rural settings have reported significant variation in vaccination rates, with higher rates in urban areas in Borno (46%) and Abuja (46%) compared to rural/semi-urban Borno (15%) and Abuja (15%) (Table 2).

**Table 2:** Summary of findings from dog demographic studies.

State	Year	Region	Setting	Human- Dog ratio	Estimated dog population	Street/free roaming	Vaccination coverage	Breed?	Reference
Bauchi	2014	NE	City	4.1:1	7,670	5,310	26.4%	Local-62.8	[119]
Borno	2007	NE	City	4.1:1-U; 3.2:1-R			46%; 15.6%	62.4	[120]
Lagos	2014	SW	City	5.6:1	1,527,718	1,427	64%		[54]
Nasarawa	2018	NC	City	6:1.1	462,586		21%	98%	[121]
Abia	2014	SE	City	7.8:1	68,121	126	47.9%	34%	[56]
FCT-Abuja	2018	NC	City	3.7:1	103,758	396	66.4%-U; 38%-R		[55]
Niger	2017	NC	City	5.4: 1	732,476		70%	60	[50]
Kwara	2012	NC	City	13:1	1,258			15	[51]

U-urban R-rural

From dog ecology studies, dogs were kept mainly for security purposes (52–98%) and were mostly native breeds, except for Abia State, where exotic breeds formed the majority (66%) [56]. Most owned dogs roamed freely. Studies reporting street counts indicated dog numbers between 126–5,310 (Bauchi, Lagos, Abuja, and Abia). Leftovers were mostly fed by the family to their dogs (44%-69%), with few allowed to scavenge or provide meals. In a study in Gwagwalada, about 56% of dog owners dumped their waste in the public space as a source of food to dogs in the community.

### Community KAP surveys towards rabies

Six studies looked at community KAP across Nigeria. These consisted of a survey of the KAP of dog owners in Taraba (2014) [57], dog meat butchers and consumers in Abia and Kaduna (2014, 2013) [58,59], children in Kaduna (2012) [60] and residents of Abuja (2018) and Lafia (2017) [61,62]. There were frequent deficiencies in knowledge of the recommended age for vaccination of dog and mode of RV transmission among dog owners and residents of Taraba and Abuja [57,61]. Only 14% and 19% of residents in urban and semi-urban areas of Lafia, respectively, indicated that they would vaccinate their dogs against rabies [63]. Education played a significant role in KAP among children attending school and residing within a learning institution [60]. Most dog meat butchers and consumers in Abia were aware of rabies, but more than 70% said they would use a nonorthodox treatment upon exposure to dog bites and not seek PEP [23]. The therapy involved burnt plants and other concoctions at the site of the bite [23]. A study in Kaduna found that dog meat consumers and processors are deficient in rabies knowledge (i.e., based on low knowledge scores reported) [59].

### Rabies in Nigerian livestock and wildlife

A total of 14 studies in our review reported livestock (n = 7) or rabies in wildlife (n = 7). One study reported a case in a captive caracal lynx and a civet cat (1978) [64]. A study on rabies in Yankari Game Reserve demonstrated the detection of RV antigens in mongoose, jackals, squirrels, hyrax and wild cats (2014)[65]. In serological studies, antibodies to four lyssaviruses, Lagos bat virus (LBV), Mokola virus (MOKV), Shimoni bat virus (SHIBV) and West Caucasian bat virus (WCBV), were detected (2010, 2020, 1990, 2014) [66–70]. Rabies in livestock has been reported in multiple species, including swine (1978) [71], cattle (2017, 2013, 2014, 2020) [72–75], sheep (2014, 2017) [74,76], goats (2014, 2018) [74,77], and a donkey (2020) [78].

## Discussion

In the absence of a national rabies control program, Nigeria remains a nation grappling with an endemic zoonosis with a high disease burden and repeated outbreaks [16,19,77]. The results of our scoping review highlight domains of rabies prevention and control that require urgent attention to alleviate the burden of disease in both human and animal populations. These studies provide a much-needed background that scopes the current understanding of rabies in the country and can be used as a baseline for planning and implementing priorities towards an effective national rabies program toward the ’zero by 30’ global target [79]. Our results indicate that most dog samples processed in canine rabies prevalence studies were from dogs meant for human consumption, purchased from dog meat markets. Dog trade for human consumption is common in some Nigerian states, namely Plateau, Akwa Ibom, Abia, Kaduna, and Kogi [19,23,80]. For example, the Plateau State is a significant hub for dog trade [33]. Dogs from different parts of Nigeria, such as in the North of the country and neighbouring countries (e.g., Niger and Chad), are transported to the Dawaki Market in Plateau State for slaughter and onward movement to various destinations within the country [81]. While dog trade for human consumption provides an essential income source to local small businesses, this market chain presents a significant risk to individuals involved in this activity. The principal risks of human RV exposure associated with dog trade for consumption result from the potentially high likelihood to be rabid. Also, dog butchers often fail to be vaccinated or use personal protective equipment (PPE) when handling dogs [23]. Indeed, two studies reported the presence of RV antibodies in dog butchers without a prior history of vaccination[24,82], suggestive of previous exposure and potential subclinical infection. These findings are of public health concern and highlight the need for health promotion interventions targeting dog butchers in Nigeria. Moreover, transportation of trade dogs between countries within the West African region was evident [34,81]. Regional transportation of dogs presents a significant concern for national rabies control efforts, mostly when dog movement occurs between relatively porous land borders. There is a need for regional collaboration and cooperation, especially with bordering countries within the West African region through joint education, coordinated vaccination campaigns and collaborative surveillance activities. Aside the public health implications of dog trade, in Nigeria, dogs meant for slaughter are often subjected to extreme cruelty[23], highlighting animal welfare concerns that require urgent attention. There is a need for awareness creation on the risk of dog trade on human health and animal welfare.

Dog bites were common across the states represented in our review, with a disproportionate risk of bites in adult males and children, along with low vaccination rates (12% - 38%) in owned dogs. Contrary to the general belief that most dogs in Africa are strays and constitute a significant challenge for rabies control, results from this study suggest that most biting dogs are owned, with low vaccination rates, consistent with a study that found only a minority (1.9%) of dogs in Africa are ownerless [83]. This finding is of great public health concern and underscores the need for public education to dog owners in Nigeria for responsible dog ownership. The low level of knowledge observed in most KAP studies and the fact that dog owners have to pay for vaccination may partly explain these findings. While studies reported reasonable compliance to rabies PEP rates among dog bite victims (64% - 92%), many people reported non-compliance, while others resort to unorthodox treatments [40,44]. Some of the examples of local treatment reported included: consuming the cooked liver of a stray dog; placing pulled hair from the dog’s neck on the bite wound [16]; use of herbal preparations and inscription of marks and tying the bite site [42]; and the use of various concoctions [23]. Low compliance with vaccination and PEP and the preference for local/traditional treatments might be responsible in part for the recorded rabies mortality. Two studies reported mortality among victims who used local therapies [16,21]. A significant deterrent to compliance is the concern that dog bite victims must pay for PEP in Nigeria, which creates an economic barrier for many exposed individuals. Unfortunately, PEP is not readily available in primary and secondary health facilities [22]. Moreover, victims in more remote, impoverished areas have to travel long distances to seek care [16]. Another element to the compliance and health-seeking behaviours towards dog bites is a limitation identified by the community KAP data. Background knowledge on rabies was highly variable across states, partly due to demographic differences, such as education level and occupation. Indeed, studies have shown that civil servants were 4.8 times more likely to have good rabies knowledge in Taraba State [57]. Children receiving formal education were more aware of rabies than those receiving informal education in Zaria [60].

Although most studies were in urban centres where access to health education is likely to be highest, our results indicate a deficiency in rabies knowledge amongst respondents [57]. If urban residents are deficient in rabies knowledge, one can only imagine rural areas where the burden is assumed to be higher. Nevertheless, very few studies were in rural settings. Those most at risk to rabies and dog bites, such as children and dog-meat butchers, were deficient in such basic knowledge. There is a need to enhance community understanding about rabies and to promote responsible dog ownership through mass enlightenment campaigns, using television and radio stations targeting school-aged children [81]. Non-conventional methods, such as pictorials, village announcements (in local languages), and community plays, can enhance rabies knowledge in rural settings. The creation of rabies clubs in primary schools can increase awareness among children [84,85].

Moreover, a significant proportion of dog bites are reported primarily to veterinary hospitals (27%), consistent with the finding in the Democratic Republic of the Congo, where 39% of dog bite victims sought care at veterinary hospitals, particularly for human PEP [86]. Targeted education is necessary on the proper reporting channel and good record-keeping practice by veterinarians in Nigeria. The ideal practice is that dog bite victims should seek PEP in a human hospital and inform veterinary clinics about the suspected dog [86]. While children and males are most at risk for bites (and correspondingly RV exposure), consistent with studies from other regions [87], veterinary hospital studies in Nigeria reported more dog bites among adults than children [38,88]. Adults may be more aware of rabies risk and the need to ascertain the offending dog’s status through quarantine and observation in a veterinary hospital following a bite. Also, parents of children victimised by a dog bite are likely to ensure that their children receive proper care in a human hospital. At the same time, adults may minimise the need for health care following a dog bite by visiting a veterinary clinic, which tends to be less crowded than human hospitals.

The reported low dog vaccination rate is consistent with prior dog ecology studies. Operationally defined in this context, applied dog ecology studies focus on estimating the dog population’s size, ownership status and access to vaccination and healthcare [55]. Our findings highlight not only the critical gap in dog owners’ rabies education, such as the need for adequate animal health [55], but also existing challenges in sustaining the delivery of optimal vaccination coverage (i.e., 70%) [79]. The low vaccination coverage (15% and 38%) reported by most studies can partly explain how vaccination campaigns are organised and resourced. Currently, dog rabies campaigns in Nigeria are rolled out in a limited number of state capitals during the annual World Rabies Day Ceremonial (September 28) [89]. These are reactive only or as part of the rabies outbreak response [79,90]. Moreover, dog owners are required to pay for dog rabies vaccinations, and the vast majority allow their dogs to roam, which makes it difficult to restrain during vaccination campaigns [91]. Other countries in Africa, such as Kenya, Namibia, and Malawi, have implemented rabies control programs through mass dog vaccination, enhanced surveillance, and rabies awareness, but have received significant international financial support, supplemented secondarily by local government’s resources[11,12]. Nevertheless, they face several logistic challenges. Nigeria should strive to source regional support for a long-term solution for rabies instead of relying solely on international donors and non-governmental organisations (NGOs). Examples in Africa, such as Kenya and Namibia, have demonstrated that national dog censuses are essential [11,12]. A first step is to quantify the dog population at risk, which allows prioritisation of locations for the deployment of canine vaccines, which could be provided free of charge to individuals [11,12]. Accessing and resourcing hard to reach communities is also a critical step for enhancing vaccine coverage supplemented by rabies health promotion and prevention interventions. Nigeria has been able to eliminate wild polio through the deployment of materials and human resources, and the utilization of innovative strategies in reaching underserved communities [92,93]. Similarly, a national rabies control program would benefit from the success recorded in polio management in Nigeria, especially in accessing rural communities with rabies vaccines and health promotion interventions.

Compared to human and dog rabies, livestock and wildlife rabies in Nigeria have been much less studied. In some countries, wildlife plays an essential role in the epidemiology of sylvatic rabies with recognised impacts on humans, dogs and other companion animals, livestock health and production, and conservation biology [94]. However, although multiple lyssavirus species have been detected in Nigeria, the role of wildlife in rabies epidemiology has been studied poorly. Rabies reported in a few wildlife species and spillover potential to/from domestic dogs has also been demonstrated [95,96]. In Nigeria, most livestock cases occur after bites from rabid dogs[73,75]. This observation suggests that control of canine rabies is likely to affect cases in livestock positively. While livestock cases are typically considered dead-end, zoonotic transmission has been reported on rare occasions [97]. A more considerable health economics impact may be to the small farmer with significant impacts on their livelihood from losing a single cow, donkey, or goat. Therefore, inter-related human health, economic and animal health impacts of dog rabies need to be investigated further.

Significant geographical heterogeneity occurs with regards to the published evidence on canine and human rabies in Nigeria. The northern states accounted for more animal rabies studies (dogs, livestock and wildlife) and fewer human rabies deaths [37,98]. Concomitantly, the southern region had a smaller number of canine rabies and dog bite studies and more human rabies deaths. The presence of NVRI, active research into rabies at Ahmadu Bello University (ABU), Zaria and cultural and religious practices in specific communities might account for this disparity in countrywide data availability and results. Individuals bitten by dogs in regions with a capacity for testing can quickly learn the diagnostic test outcome and seek care, unlike people located further away. Besides, more veterinary schools in the northern region (n = 6) compared to the southern part (n = 4) [99], might also contribute to the fewer studies in the south. For example, south-south states without an accredited veterinary school had the lowest reported prevalence of dog rabies. Also, Rivers, a state in the South-South geopolitical zones of Nigeria with a population of 5,198,716 as of the 2006 population census, has only one veterinarian in the government service [14]. Furthermore, victims of rabies in the northern region of Nigeria are more likely to be buried due to local religious beliefs of immediate burial practice by Muslims that form the ethnic majority in the north of the country, thereby precluding diagnosis. Taken together, these findings highlight the need to enhance surveillance capacity, especially in the southern regions, through supporting the provision of rabies diagnostic facilities for the prompt processing of samples and RV characterization. Integrated dog bite management can improve the early detection of dog rabies, as shown in Tanzania [100]. Local inclusion of dog bite occurrence by disease surveillance and notification officers (DSNOs), with related community surveillance tools can help in rabies/dog surveillance. Empowering private veterinarians and those in rural areas with decentralized, rapid point-of-care diagnostic abilities, such as the dRIT—with high sensitivity and specificity [101] as well as other options, can improve regional rabies surveillance. Veterinary and human hospitals should share dog bite information so that veterinarians can ascertain the biting dog’s status and that the human victims can receive prompt PEP.

### Limitations

The findings of this study should be interpreted in light of several limitations. First, the low number of studies in each subject category under review, the geographical and temporal range of available studies (1978–2020) and the incomplete data on dog information such as breed, vaccination status, and detailed information of victims of dogs bite and lack of laboratory confirmation, precluded a more comprehensive quantitative analysis of rabies epidemiology in Nigeria. Second, there was a strong geographical bias of studies on dog rabies. Only a handful of studies approached rabies from a One Health perspective, thereby impeding a more thorough understanding of rabies in Nigeria for effective prevention and control. Third, our review’s available studies did not allow a comprehensive evaluation of the role of risk factors in RV transmission and highlight areas most in need of interventions. Finally, we relied on data from published articles and the grey literature, and the availability of national rabies data (human and animal) could have supported a more robust analysis.

## Conclusions

Dog trade for human consumption presents significant public health risks, particularly to community members such as dog meat butchers and consumers involved in the market chain. Rabies risk, coupled with welfare concerns, demonstrates the need for continued advocacy towards legislation prohibiting the dog trade in Nigeria and elsewhere. Dog bites in Nigeria are reported to both human and veterinary hospitals, with disproportionately affected children and males. Some victims (dog bite) use traditional medicine in place of PEP. The high-risk groups identified in this review (children, butchers, and adult males) and the general public need to be educated on response to dog bite exposure to reduce the impact of this invariable fatal but preventable disease. Most biting dogs are owned and present with very low vaccination rates. There is a need for mandatory dog vaccination and implementation of a national rabies program to attain the WHO recommended vaccination coverage of at least 70%. There were fewer studies on rabies in livestock in Nigeria, and all cases were from a bite from a rabid dog. Controlling dog rabies will impact the occurrence of rabies in livestock, and there is a need to investigate rabies health economic impact on smallholder farmers in Nigeria. There was a significant geographical heterogeneity concerning dog rabies, dog bite, and human rabies, with more dog rabies studies in the northern region and more human rabies and cases in the southern part. This observation appears to be due mainly to active research into rabies at the Northern University (ABU), and the presence of national rabies diagnosis is in the northern region. Establishing rabies diagnostic centres in the country’s six geopolitical zones, local inclusion of dog bite occurrence by DSNOs, with related community surveillance tools, can help in rabies/dog surveillance. While canine rabies is the priority, pathogen discovery and laboratory-based surveillance for wildlife rabies should be enhanced. Private veterinarians and those in rural areas should be empowered with a decentralized, rapid point-of-care diagnostic ability, such as the dRIT, which is comparable to FAT for animal diagnosis, with an added advantage of use under field conditions [102]. Employment of veterinarians in states like Rivers State without a veterinary presence (public servants) will go a long way towards improving local rabies surveillance.

## Supporting information

S1 Table Summary of studies reporting dog bite incidents and rabies deaths across the Nigerian States. (DOCX)
