# Supplementary material for: Rabies epidemiology, prevention and control in Nigeria: Scoping progress towards elimination
Source: PLoS Negl Trop Dis. 2021 Aug 16;15(8):e0009617. doi: 10.1371/journal.pntd.0009617 (PMC8389847; doi:10.1371/journal.pntd.0009617)
Supplement: S1 Table — (DOCX) [file pntd.0009617.s001.docx]

Table 2. Summary of studies reporting dog bite incidents and rabies deaths across the Nigerian States.

|  | **State** | **Where the study was carried out** | **Year** | **Total bites** | **Rabies deaths**  **(%=# deaths/bites/ rabies)** | **Gender** | **Proportion of children** | **Number of bitten patients that received PEP** | **Dog characteristics** | **Traditional care** | **Proportion of unprovoked bite** | **Proportion of dogs vaccinated** | **Reference** |
| --- | --- | --- | --- | --- | --- | --- | --- | --- | --- | --- | --- | --- | --- |
| 1 | Ebonyi | Human | 2018 | 74 | 1(1.4%) | M-36  F-38 |  | 87%(64/74) | owned dog-51  Stray -23 |  | 81% (60/74) | 38% (23/74) | [1] |
| 2 | Ekiti | Human | 2017 | 84 | 6(7.1%) | M-60  F-24 | 60.7% | 92%(78/84) | Owned – 66  Stray - 18 | *60%(50/84) | 71.4% (60/84) | 1.2% (1/84) | [2] |
| 3 | Ekiti | Veterinary | 2012-2017 | 44 | 12(27.3%) |  |  |  |  |  |  |  |  |
| 4 | Kaduna | Human | 2000-2010 | 81 | 2(2.4%) | M-67  F-15 | 55.6% (45/81) | 87.7% (71/81) | Owned- 39  Stray - 42 |  |  |  | [3] |
| 5 | Enugu | Human | 2004-2013 | 149 | 6(4%) | M-83  F-66 | 55.7%(83/149) |  | Owned-63  Stray-86 | 54.4%(81/149) |  | 12.1% (18/149) | [4] |
| 6 | Lagos | Human | 2006-2011 | 196 | 1(0.5%) | M-92  F-87  N/A-17 | **35.2%(69/196) |  |  |  |  | 79.6% (156/196) | [5] |
| 7 | Plateau | Human | 2007-2016 | \| 884 \| \| --- \| | 0 | M-472  F-412 | 46.5% (411/884) |  |  |  |  |  | [6] |
| 8 | Plateau | Veterinary | 2009-2010 | 247 | 6(2.4%) |  | 13.4%(33/247) |  | Owned- 223  Stray-24 |  |  | 5.3% (13/247) | [7] |
| 9 | Abia | Veterinary | 2014 | 215 | 0 | M-135  F-80 | 11.6%(25/215) |  | Owned-  Stray - |  |  | 10.2% (22/215) | [8] |
| 10 | Osun | Human | 1996-1997 | 174 | 2(1.1%) | M-109  F-65 | 100% |  | Owned-65  Stray-109 |  | 96.6% (168/174) |  | [9]  [10] |
| 11 | Kwara | Human | 2000 | 39 | 4(10.3%) | M-28  F-11 | 100% |  | Owned-16  Stray- 23 |  | 38.5% (15/39) | 28.2% (11/39) | [11] |
| 12 | Kano | Human | 2010 | 44 | 5(11.4%) | M-38  F-6 | 100% |  | Owned-16  Stray-28 |  | 59.1% (26/44) | 30% (12/44) | [12] |
| 13 | Plateau |  | 2006-2010 | 713 | 299 (41.9%) | M-404  F-309 | 52.9%(377/713) | 99.6% | Owned-337  Stray - 376 |  |  | 2.5% (18/713) | [13] |
| 14 | Kwara | Human | 2002-2008 | 152 | 17(11.2%) | M-73  F-79 | 32.2%(49/152) |  |  |  |  |  | [14] |
| 15 | Enugu | Human | 2014 | 127 | 5(4%) |  | 46.5% |  |  |  |  |  | [15] |
| 16 | Edo | Human | 1997-2009 | 143 | 1(0.7%) | M-126  F-17 | 11.9%(17/143) | 71% | Owned-63 | 0.7%(1/143) |  |  | [9] |
| 17 | Cross River | Human | 2009 | 11 | 0 | M-6  F-5 | 18.2%(2/11) |  |  |  | 36.4% (4/11) |  | [16] |
| 18 | Cross River | Veterinary | 2001-2011 | 183 | 0 | M-95  F-88 | 56.8%(104/183) |  |  |  |  | 43.9% (687/1562) | [17] |
| 19 | Calabar | Human | 2013 | 10 | 10(100%) | M-8  F-2 | 50% (5/10) | 0 | Owned-8  Stray-2 | 10%(1/10) | 20%(2/10) | 0 | [18] |
| 20 | Edo | Human | 2014 | 47 | 4(9%) | M-30  F-17 | 63.8(30/47) | 95.7%(45/47) |  | 6.6%(3/47) |  |  | [19] |
| 21 | Imo | Human | 2005-2014 | 436 | 17(4%) | M-237  F-199 | 51.2%(223/436) |  |  |  |  | 54.5% (5595/10264) | [20] |
| 22 | Ekiti | Veterinary | 2019 | 44 | 12(37.5%) |  |  |  |  |  |  |  | [21] |
| 23 | Sokoto | Human | 1980-1998 | 1361 | 11(0.8%) | M-6  F-5 | 63.6%(866/1361) |  |  |  |  |  | [22] |
| 24 | Zamfara | Veterinary | 2019 | 1 | 1(100%) |  |  |  |  |  |  |  | [23] |
| 25 | Kaduna | Veterinary | 2019 | 4 | 2(50%) | M-1  F-1 | 0 | 50%(2/4) | Owned-1  Stray-1 | 25%(1/32) | 100% | 0 | [24] |
| 26 | Abia | Veterinary | 2004-2013 | 110 | 7(6.4%) | M-59  F-51 | 28.2% |  | Owned-90  Stray-20 | 0.9%(1/110) |  | 9.1% (10/110) | [25] |
| 27 | Niger |  | 2006-2013 | 223 | 0 | M-149  F-74 | 46.2%(103/223) |  |  |  |  |  | [26] |
| 28 | Abia | Veterinary | 2017 | 1 | 1(100%) | M-1 | 0 | 0 | Owned-1 | 100% | 100% | 0 | [27] |
| 20 | Zamfara | Veterinary | 2018 | 1 | 1(100%) |  |  |  |  |  |  |  | [28] |
| 30 | Ogun | Human | 2013-2017 | 318 | 2(1.1%) | M-178  F-140 | 49.3%(157/318) |  |  |  |  |  | [29] |

1. Omoke N, Chukwueloka Onyemaechi N. Incidence and pattern of dog bite injuries treated in the emergency room of a teaching hospital South East Nigeria. African Journal of Medical and Health Sciences. 2018;17(1):35-40. doi: 10.4103/ajmhs.ajmhs_59_17.

2. Ogundare EO, Olatunya OS, Oluwayemi IO, Inubile AJ, Taiwo AB, Agaja OT, et al. Pattern and outcome of dog bite injuries among children in Ado-Ekiti, Southwest Nigeria. Pan Afr Med J. 2017;27:81. Epub 2017/08/19. doi: 10.11604/pamj.2017.27.81.7360. PubMed PMID: 28819502; PubMed Central PMCID: PMCPMC5554624.

3. Abubakar S, Bakari A. Incidence of dog bite injuries and clinical rabies in a tertiary health care institution: A 10-year retrospective study. Annals of African Medicine. 2012;11(2):108-11. doi: 10.4103/1596-3519.93534.

4. Eke C, Omotowo I, Ukoha O, Ibe B. Human rabies: Still a neglected preventable disease in Nigeria. Nigerian Journal of Clinical Practice. 2015;18(2):268-72. doi: 10.4103/1119-3077.151064.

5. Hambolu SE, Dzikwi AA, Kwaga JK, Kazeem HM, Umoh JU, Hambolu DA. Rabies and dog bites cases in lagos state Nigeria: a prevalence and retrospective studies (2006-2011). Glob J Health Sci. 2013;6(1):107-14. Epub 2014/01/01. doi: 10.5539/gjhs.v6n1p107. PubMed PMID: 24373270; PubMed Central PMCID: PMCPMC4825396.

6. Konzing L, Kwaga JKP, Kia GSN, Kazeem HM. A retrospective study of dog bite cases reported to some hospitals in Plateau State, Nigeria. Sokoto Journal of Veterinary Sciences. 2019;17:56-61.

7. Bata SI, Dzikwi AA, Ayika DG. Retrospective Study of Dog Bite Cases Reported to Ecwa Veterinary Clinic, Bukuru, Plateau State, Nigeria. Science World Journal. 2011;6:17-9.

8. Richard OG, Umoh U, Adamu DA. Cases of Dog Bite in Aba, Abia State Nigeria and Its Public Health Significance. International Journal of Tropical Disease & Health. 2014;4:1097-103.

10. Oginni FO, Akinwande JA, Fagade OO, Arole GF, Odusanya SA. Facial dog bites in Southwestern Nigerian children: an analysis of eight cases. Trop Doct. 2002;32(4):239-40. Epub 2002/10/31. doi: 10.1177/004947550203200423. PubMed PMID: 12405313.

11. Ojuawo A, AhdulKareem A. Dog bite in children in Ilorin. Sahel Medical Journal. 2000;3(1):33-6.

12. Adeleke SI. Impact of dog bite in Kano city a retrospective study. Nigerian Journal of Clinical Practice. 2010;13:67.

13. Aworh MK, Nwosuh C, Ajumobi O, Okewole PA, Okolocha EC, Akanbi B, et al. A Retrospective Study of Rabies Cases Reported at Vom Christian Hospital, Plateau State, Nigeria, 2006@ 2010. Nigerian Veterinary Journal. 2011;32:366-70.

14. Olugasa BO, Aiyedun J, Akingbogun AA. Identification of geographic risk factors associated with clinical human rabies in a transit city of Nigeria. Epizootiol Anim Health West Africa. 2009;5:43-52.

15. Olugasa BO, Okeke OS, Ishola OO. Geographic access to street food sources for dogs and its association with spatial patterns of animal bite injuries in Enugu, Nigeria, 2005-2011. Afr J Med Med Sci. 2014;43 Suppl:79-86. Epub 2014/12/01. PubMed PMID: 26949784.

16. Asuquo M, Ndifon W, Ugare G, Mwankon J. Prevalence Of Dog Bites In A Rural Community: A 15 Year Review Of Cases In Okoyong, Cross River State, Nigeria. Journal of Community Medicine. 2009;2:65-9.

17. Isek TI, Umoh J, Dzikwi AA. A retrospective study of dog bite occurrence and anti-rabies vaccination of dogs in a State Veterinary Hospital in Ogoja, Cross River State, Nigeria. Vet Ital. 2019;55(2):163-8. Epub 2019/07/06. doi: 10.12834/VetIt.150.431.5. PubMed PMID: 31274178.

18. Ekanem EE, Eyong KI, Philip-Ephraim EE, Eyong ME, Adams EB, Asindi AA. Stray dog trade fuelled by dog meat consumption as a risk factor for rabies infection in Calabar, southern Nigeria. African health sciences. 2013;13(4):1170-3. Epub 2014/06/19. doi: 10.4314/ahs.v13i4.44. PubMed PMID: 24940348; PubMed Central PMCID: PMCPMC4056491.

19. Iyalomhe G, Iyalomhe S. Dog bite and clinical rabies in a suburban hospital in Nigeria: a 20-year retrospective study of the prevalence and treatment with anti-rabies vaccine. World J Pharm Res. 2014;4(1):113-21.

20. Anosike IG. Trends of dog bite, clinical human rabies and anti-rabies vaccination coverage in imo state, Nigeria (2005-2014): University of Ibadan 2016.

21. Adebayo J, Ojo V, Ogundipe G, Nguku PM. Evaluation of Animal Rabies Surveillance System, Ekiti State, Nigeria, 2012-2017. Online J Public Health Inform. 2019;11(1):e326. doi: 10.5210/ojphi.v11i1.9784. PubMed PMID: PMC6606125.

22. Ahmed H, Chafe UM, Magaji AA, Abdulqadir A. Rabies and dog bite in children: a decade of experience in Sokoto, Nigeria. Sokoto Journal of Veterinary Sciences. 2000;2:2-10.

23. Ahmad I, Kudi CA, Abbas M, Yakubu Y, Muhammad U, Salisu MD. Human death from suspected rabid dog bite in Zamfara State, Nigeria. Sokoto Journal of Veterinary Sciences. 2019;16:92-5.

24. Audu SW, Mshelbwala PP, Jahun BM, Bouaddi K, Weese JS. Two fatal cases of rabies in humans who did not receive rabies postexposure prophylaxis in Nigeria. Clin Case Rep. 2019;7(4):749-52. doi: 10.1002/ccr3.1972. PubMed PMID: 30997078.

25. P.PMshelbwala, VB M, M A, U.C N, Nwakocha Q, AB O. Retrospective study of dog bite cases reported to zonal veterinary clinic, umuahia, Abia state, Nigeria. Journal of Experimental Biology and Agricultural Sciences. 2013;1(4):3.

26. Garba A, Umoh J, Kazeem H, Dzikwi A, Yahaya M, Zaharadeen A, et al. Hospital Records (2006-2013) of Dogbite Cases and Laboratory Confirmation of Dog Rabies in Niger State, Nigeria. International Journal of Animal and Veterinary Advances. 2014;6(2):87-91.

27. Mshelbwala PP, Weese JS. Rabies in the developing world: challenges & prospects. 2017.

28. Ahmad I, Kudi C, Abbas M, Yakubu Y, Muhammad U, Salisu M. Human death from suspected rabid dog bite in Zamfara State, Nigeria. Sokoto Journal of Veterinary Sciences. 2018;16(4):92-5.

29. Mshelbwala PP, Adegbite O, Bamiselu O, Esu I, Shinkafi I, Adeyemi B, et al. One-health approach to rabies exposure surveillance within Ogun State, Nigeria: evidence of a limited collaboration between human and veterinary services. The Pan African medical journal. 2019;10.
